# Ginseng Purified Dry Extract, BST204, Improved Cancer Chemotherapy-Related Fatigue and Toxicity in Mice

**DOI:** 10.1155/2015/197459

**Published:** 2015-04-07

**Authors:** Hyun-Jung Park, Hyun Soo Shim, Jeom Yong Kim, Joo Young Kim, Sun Kyu Park, Insop Shim

**Affiliations:** ^1^Department of Science in Korean Medicine, Graduate School, College of Korean Medicine, Kyung Hee University, 26 Kyunghee-daero, Seoul 130-701, Republic of Korea; ^2^Green Cross Health Science Co., Ltd., 474 Dunchon-daero, Jungwon-gu, Sungnam, Gyeonggi-do 462-806, Republic of Korea

## Abstract

Cancer related fatigue (CRF) is one of the most common side effects of cancer and its treatments. A large proportion of cancer patients experience cancer-related physical and central fatigue so new strategies are needed for treatment and improved survival of these patients. BST204 was prepared by incubating crude ginseng extract with ginsenoside-*β*-glucosidase. The purpose of the present study was to examine the effects of BST204, mixture of ginsenosides on 5-fluorouracil (5-FU)-induced CRF, the glycogen synthesis, and biochemical parameters in mice. The mice were randomly divided into the following groups: the naïve normal (normal), the HT-29 cell inoculated (xenograft), xenograft and 5-FU treated (control), xenograft + 5-FU + BST204-treated (100 and 200 mg/kg) (BST204), and xenograft + 5-FU + modafinil (13 mg/kg) treated group (modafinil). Running wheel activity and forced swimming test were used for evaluation of CRF. Muscle glycogen, serum inflammatory cytokines, aspartic aminotransferase (AST), alanine aminotransferase (ALT), creatinine (CRE), white blood cell (WBC), neutrophil (NEUT), red blood cell (RBC), and hemoglobin (HGB) were measured. Treatment with BST204 significantly increased the running wheel activity and forced swimming time compared to the control group. Consistent with the behavioral data, BST204 markedly increased muscle glycogen activity and concentrations of WBC, NEUT, RBC, and HGB. Also, tumor necrosis factor-*α* (TNF-*α*) and interleukin-6 (IL-6), AST, ALT, and CRE levels in the serum were significantly reduced in the BST204-treated group compared to the control group. This result suggests that BST204 may improve chemotherapy-related fatigue and adverse toxic side effects.

## 1. Introduction

Colorectal cancer accounts for almost 50,000 deaths each year in the United States and remains the third leading cause of cancer-related mortality [1]. While surgical resection remains the gold standard of treatment for localized disease, chemotherapy combinations with oxaliplatin, irinotecan, and 5-fluorouracil (5-FU) have led to significant improvements in survival at all stages [2–5]. However, these chemotherapies negatively impact physical and social function including cancer-related physical and central fatigue. Actually, 80–96% of chemotherapy patients are estimated to have experienced cancer related fatigue (CRF) that is characterized by a persistent and unusual sense of tiredness, weakness, need for rest, and decreased physical performance [6]. Chemotherapy is known to increase concentrations of proinflammatory cytokines such as TNF-*α*, IL-6, and IL-1*β* in cancer patients. These cytokines can influence appetite, pain, sleep disturbance, anorexia, or anemia which may interact to produce fatigue, accompanied by hepatotoxicity, nephrotoxicity, or hematosuppression in cancer patients undergoing chemotherapy [7–12].

Fatigue can have a profound negative impact on a person's ability to function and quality of life [13]. In this regard, more and more attention has been paid to factors correlated with CRF [14]. So, new strategies are needed for CRF treatment without side effect.

Growing evidence shows that natural product is one of the most promising new multidisciplinary approaches for cancer therapy [15–18]. Many studies already reported the anticancer effect of ginseng [19–23]. Ginsenosides are known to be major active ingredients underlying the pharmaceutical action of ginseng. Ginsenosides are a diverse group of steroidal saponins showing the ability to target a vast range of tissues. With the recent development in the separation and analysis technology, the chemical makeup of 30 ginseng saponins has been identified up to date. Some of ginsenosides have been reported to produce anticancer effects. For example, Rh_2_ markedly inhibited tumor cell growth and proliferation of various cultured cancer cells and can influence apoptosis [24–28]. Rg_3_ also has angiosuppressive effects and antitumor properties [29]. Previously, Seo et al. reported that a fermented ginseng extract BST204, containing 10.9% of Rg_3_ and 7.2% of Rh_2_, reduced p70 S6 kinase activation on RAW 264.7 cell lines [30].

Based on the previous reports [31–43], it is possible that BST204 may reduce the severity of the common symptoms of side effects including fatigue and toxicity. However, there has been no report of BST204 on CRF and adverse toxicity such as hepatotoxicity, nephrotoxicity, or hematosuppression in 5-FU-induced CRF animal model. In the present study, we explored the effect of BST204 on 5-FU-induced CRF and the mechanisms of its action were investigated.

## 2. Materials and Methods

### 2.1. Cell Culture

The HT-29 human colorectal cancer cells (KCLB 30038) were kindly provided by Korea Cell Line Bank from Republic of Korea. The cells were grown in Dulbecco's modified Eagle's medium (DMEM) supplemented with 10% (v/v) heat-inactivated fetal bovine serum under a 5% CO_2_/95% humidified air at 37°C (Sanyo, MCO-15AC, Japan), and the cells were fed on alternative days. The cells were subcultured every 3-4 d and the medium was changed every 2-3 days.

### 2.2. Animals

Five-week-old female Balb/c-nu/nu mice weighing 18–20 g each were purchased from Harlan Laboratories (Kyungki-do, Korea). The animals were allowed to acclimatize themselves for at least 7 days prior to the experiment. The animals were housed in individual cages under light-controlled conditions (12/12 hr light/dark cycle) and at 23°C room temperature. Food and water were made available ad libitum. All the experiments were approved by the Kyung Hee University Institutional Animal Care and Use Committee (A-BST204-20120101). Also, this experimental protocol was approved by an institutional review committee for the use of human or animal subjects or that procedures are in compliance with at least the Declaration of Helsinki for human subjects, or the National Institutes of Health Guide for Care and Use of Laboratory Animals (Publication number 85-23, revised 1985), the UK Animals Scientific Procedures Act 1986 or the European Communities Council Directive of 24 November 1986 (86/609/EEC).

### 2.3. Cancer Related Fatigue (CRF) Animal Model and Drug Treatment

HT-29 (5 × 10^6^ cells/0.2 mL/mouse) cultured in DMEM was subcutaneously injected into the right flanks of the mice. All inoculated, except phosphate buffered saline-injected normal, mice formed a tumor within 14 days. Tumor volume was measured with a digital electric caliper and calculated by the following formula: (width in mm)^2^  ×  (length in mm)/2. The treatment started when the tumor size reached 100~150 mm^3^.

The mice were randomly divided into the following groups: the naïve normal (Normal), the HT-29 cell inoculated + saline treated group (10 mL/kg) (xenograft), xenograft + 5-FU + vehicle (2.5% EtOH, 2.5% Tween 20 in DW, 10 mL/kg) treated group (control), xenograft + 5-FU + BST204-treated group (100 and 200 mg/kg) (BST204), and xenograft + 5-FU + modafinil (13 mg/kg) treated group (modafinil).

Modafinil and 5-FU were dissolved in saline. Also, BST204 was dissolved in 2.5% EtOH, 2.5% Tween 20 in DW. Mice were weighed immediately prior to injection and were given the 5-FU (Choongwea Pharmaceutical, Korea) treatment in proportion to body weight (30 mg/kg) through intraperitoneal (i.p.) injections 3 times a week for 28 days for induction of CRF. A fatigue-mitigating drug, modafinil, is used as positive control for the purpose of comparison [44, 45].

**Figure 1:**
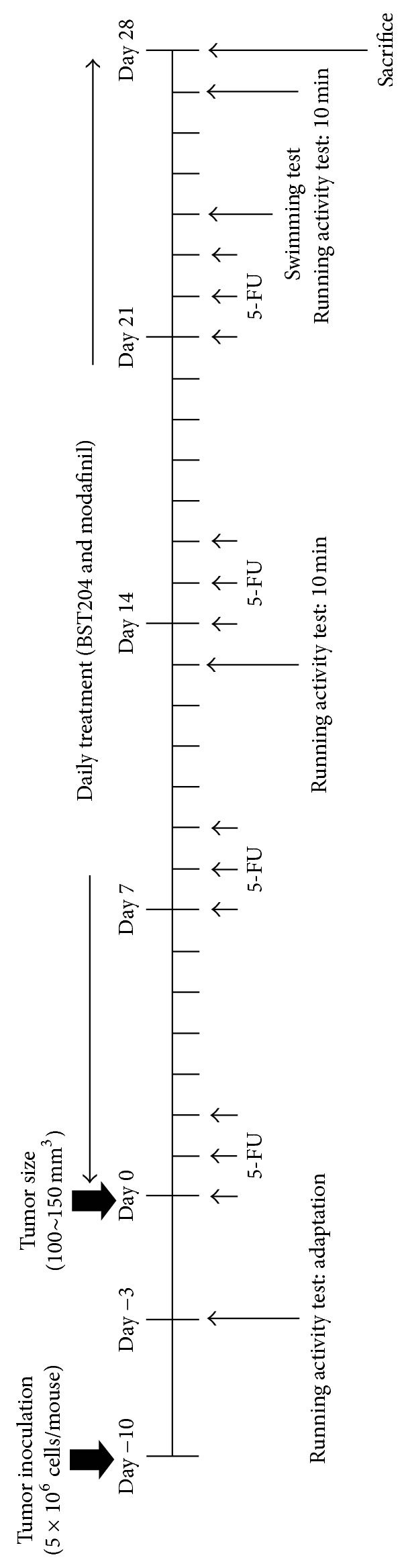


Modafinil (13 mg/kg, Choongwea Pharmaceutical, Korea), BST204 (Green Cross Health Science (GCHS), batch number BST204-P-5012 (Rg_3_: 10.9%, Rh_2_: 7.2%), Korea), or saline was administrated p.o. every day for 28 days. Tumor volume was evaluated twice a week after drug treatment [2, 3]. Experimental schedule is depicted in Figure 1.

### 2.4. Preparation of Fermented Ginseng Extract, BST204

BST204 was gifted from GCHS Co. Ltd. (Seongnam, Korea). It was manufactured according to a patent technology and the previously reported study [20]. Briefly, the harvested ginseng was extracted with ethanol repeatedly followed by reaction with an enzyme containing ginsenoside-*β*-glucosidase. After acid hydrolysis of the residue, the reactant was purified with HP-20 resin followed by washing out with distilled water and finally 95% ethanol. Ninety-five % of ethanol fractions, consisting Rg_3_ and Rh_2_, were concentrated and were designated as BST204.

### 2.5. Running Wheel Activity

Running wheel activity was used to estimate the voluntary activity of the animals as the parameter of CRF. All animals were adapted to voluntary running wheel activity test prior to drug treatment for 5 min. Mice were tested for wheel running activity on 13th and 27th day after drug treatment for 10 min. During assessing the VWRA, all mice were acclimated to the testing room and examined running wheel activity for 10 minutes and calculated in running. The apparatus was placed in a darkened, light and sound attenuated and ventilated testing room. The voluntary running wheels were equipped with counters that recorded distance traveled and time spent running (Jeungdo B&P, Seoul, Korea). Revolutions of the wheel were counted and recorded for 10 min. Running distance was measured on 27th day after first 5-FU treatment. Running distance was calculated as follows: ∗Running distance = circumference (31 cm × 3.14) × number of wheels/10 min.

### 2.6. Forced Swimming

A transparent water bath (500 × 500 × 400 mm), which maintained the designated temperature and water level, was filled with warm water of 23 ± 1°C and the animals were forced to swimming test on the 24th day. The apparatus was placed in a darkened, light and sound attenuated and ventilated testing room. At the end of treatment with 5-FU, the swimming test was examined in mice for 10 minutes. The total swimming time was recorded right after they were judged to be unable to swim due to total exhaustion, assessed when mice failed to rise to the surface of water for breath within a 10 s period. Data showed percentage of normal. The observers were blinded to drug treatments in behavioral tests. Mice were forced to swim until fatigue, defined as failure to rise to the surface of the water to breathe within an 8-s period. The time until fatigue was recorded [46].

### 2.7. Biochemical Analysis of Blood

On day 17 of experiment from 5-FU treatment, blood was collected from the orbital plexus. Blood samples were centrifuged at 10,000 g for 10 min at 4°C. Separated serum was transferred to another Eppendorf tube. Level of hepatotoxicity (ALT, AST, and Cre) was analyzed using the Serum Chemistry Kit (HITACHI 7180, Japan).

After sacrifice, blood was collected by a syringe from the abdominal aorta and immediately transferred into K2EDTA tubes (BD Microtainer, USA). White blood cell (WBC), neutrophil (NEU), red blood cell (RBC), hemoglobin (HGB), IL-6 and TNF-*α* were measured and analyzed by ADVIA 2120 (SIEMENS, USA). Biomarkers were measured through Elisa kit of the Bioassay System (Bioassay system, CA, USA Koma Biotech, CAS# number K0331186P, CAS# number K0331230P, Seoul, Korea). The detection was made through the ELISA Reader (Bio-Rad 680, CA, USA).

### 2.8. Glycogen Measurement

At the end of drug treatment, mice were fasted for 12 h prior to obtain fasted blood and muscle harvest. After sacrifice, 0.1 g of femoral muscles tissue was extracted and placed in a 1 mL PRO-PREPTM protein extraction solution homogenized by homogenizer (IKA T10 basic). The tissue samples were centrifuged at 20,000 ×g for 10 min at 4°C. The supernatant was transferred to another tube and was analyzed by ELISA kit of the Bioassay System. Absorbance was measured at 540 nm in a microplate reader (Bio-Rad 680, CA, USA).

### 2.9. Statistical Analysis

The values of experimental results were expressed as mean ± S.E.M. Group differences were analyzed by analysis of variance (ANOVA) followed by the LSD post hoc test. In all instances, values of *P* < 0.05 were considered to be significant. Analyses were performed using SPSS statistical software (version 18.0) for Windows.

## 3. Results

### 3.1. Running Wheel Activity

**Figure 2:**
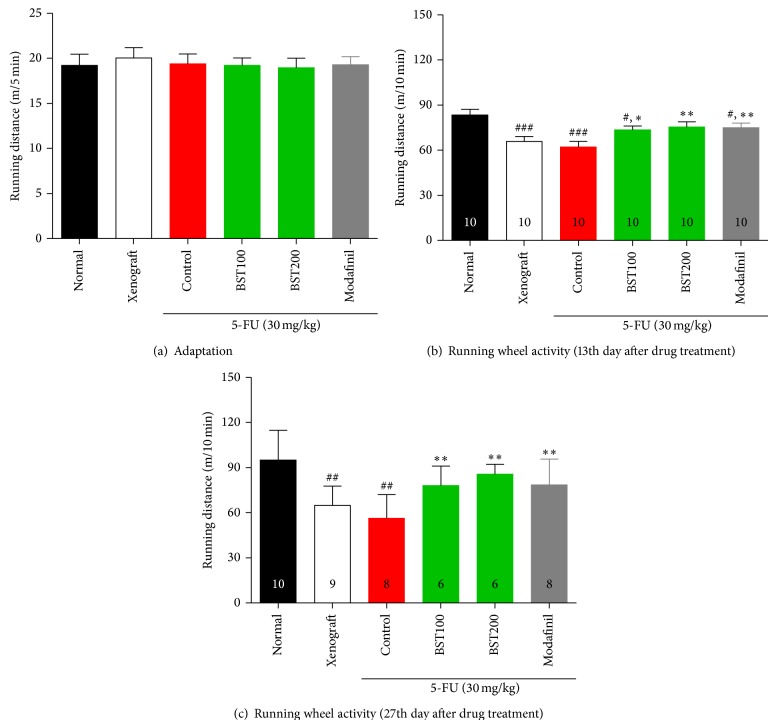
Voluntary running wheel activity during three days recorded before drug treatment as a baseline value (a), the 13th day (b), and the 27th day after drug treatment (c). Each value represents the mean ± S.E.M. ^#^ *P* < 0.05, ^##^ *P* < 0.01, and ^###^ *P* < 0.001 compared to the normal and ^*^ *P* < 0.05, ^**^ *P* < 0.01 compared to the control group. Naïve normal (normal); HT-29 cell inoculated + saline treated group (xenograft). Xenograft + 5-FU + vehicle treated group (control); xenograft + 5-FU + BST204-treated group (BST204). Xenograft + 5-FU + modafinil treated group (modafinil).

All mice exposed to a voluntary running wheel test on the 13th day (Figure 2(a); *F*_5,54_ = 5.1, *P* < 0.001), 27th day (Figure 2(b); *F*_5,41_ = 7.2, *P* < 0.001). As shown in Figures 2(a) and 2(b), running wheel activity in the control group was significantly decreased compared to the normal group (13th day: *P* < 0.001, 27th day: *P* < 0.01). However, a significant increase in running wheel activity was observed in BST204-treated or modafinil treated mice, compared to only 5-FU treated control mice. Increased running wheel activities of animals were considered as decreased physical fatigue.

### 3.2. Forced Swimming

**Figure 3:**
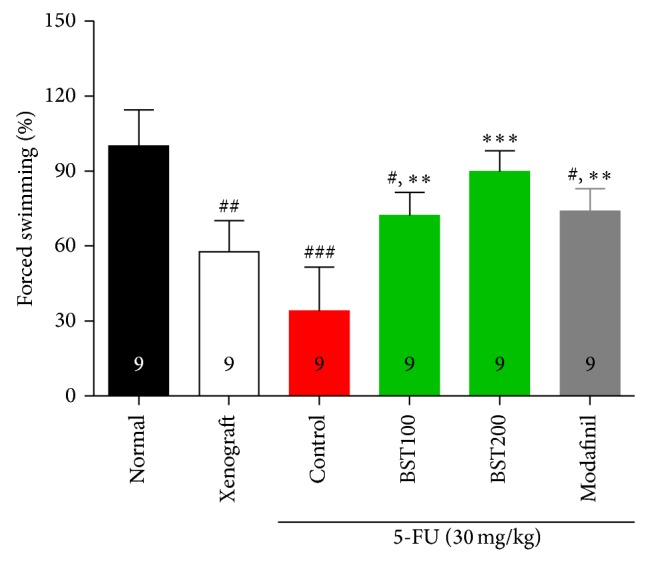
The effects of BST204 and modafinil with 5-FU treatment on forced swimming test. Each value represents the mean ± S.E.M. ^#^ *P* < 0.05, ^##^ *P* < 0.01, and ^###^ *P* < 0.001 compared to the normal and ^**^ *P* < 0.01, ^***^ *P* < 0.001, compared to the control group. Naïve normal (normal); HT-29 cell inoculated + saline treated group (xenograft). Xenograft + 5-FU + vehicle treated group (control); xenograft + 5-FU + BST204-treated group (BST204). Xenograft + 5-FU + modafinil treated group (modafinil).

The swimming test: the ability of mice to cope with a physical fatigue was evaluated (*F*_5,48_ = 7.5, *P* < 0.001). As shown in Figure 3, the animals displaying increased swimming periods were considered as decreased physical fatigue. The control group showed decreased swimming time compared to the normal group in the FST (*P* < 0.001). Also, the BST204-treated or modafinil treated mice showed increase of swimming time during 10 min the test than that of the control (BST100, *P* < 0.01; BST200, *P* < 0.001).

### 3.3. Glycogen Measurement and Biochemical Analysis of Blood

**Figure 4:**
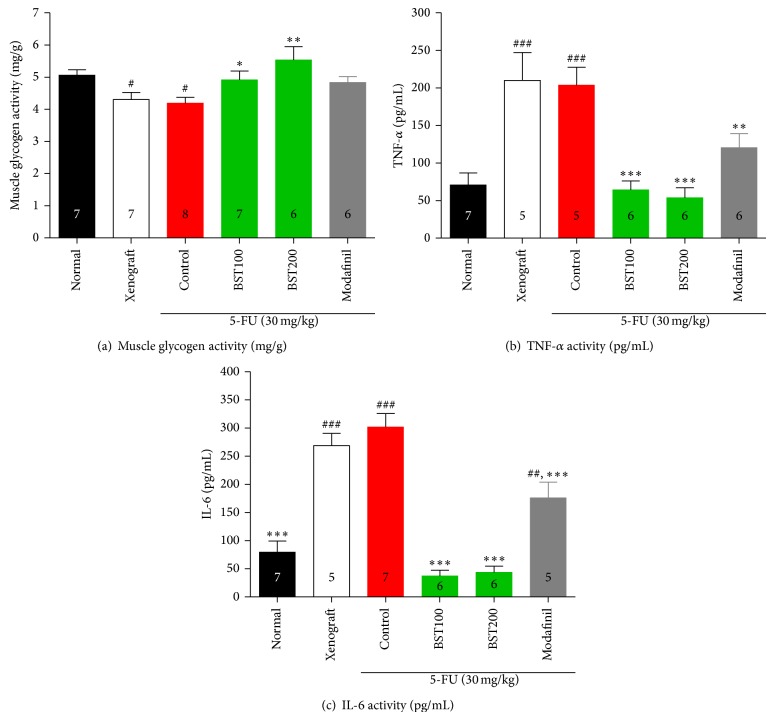
The effects of BST204 and modafinil with 5-FU treatment on glycogen synthesis (a), TNF-*α* (b), and IL-6 (c). Each value represents the mean ± S.E.M. ^#^ *P* < 0.05, ^##^ *P* < 0.01, and ^###^ *P* < 0.001 compared to the normal and ^*^ *P* < 0.05, ^**^ *P* < 0.01, and ^***^ *P* < 0.001 compared to the control group. Naïve normal (normal); HT-29 cell inoculated + saline treated group (xenograft). Xenograft + 5-FU + vehicle treated group (control); xenograft + 5-FU + BST204-treated group (BST204). Xenograft + 5-FU + modafinil treated group (modafinil).

On the last day of the experiment, the femoral muscle was examined in order to estimate the amount of glycogen in the body, which greatly influences the amount of fatigue felt by the individual or animal. The levels of muscle glycogen were significantly different among the groups (Figure 4(a); *F*_5,35_ = 4.0, *P* < 0.01). The level of glycogen in the control group was significantly decreased, compared to the normal group (*P* < 0.05). However, treatment of BST204 resulted in markedly increased levels of glycogen compared to the control group (BST100, *P* < 0.05; BST200, *P* < 0.01). The serum levels of cytokines were significantly different among the groups (Figure 4(b), TNF-*α*, *F*_5,29_ = 11.2, *P* < 0.001; Figure 4(c), IL-6; *F*_5,30_ = 35.0, *P* < 0.001). The serum levels of proinflammatory cytokines were significantly increased in the control group compared to the normal group. However, the treatment of BST204 or modafinil markedly decreased levels of cytokines compared to the control groups (*P* < 0.001).

**Figure 5:**
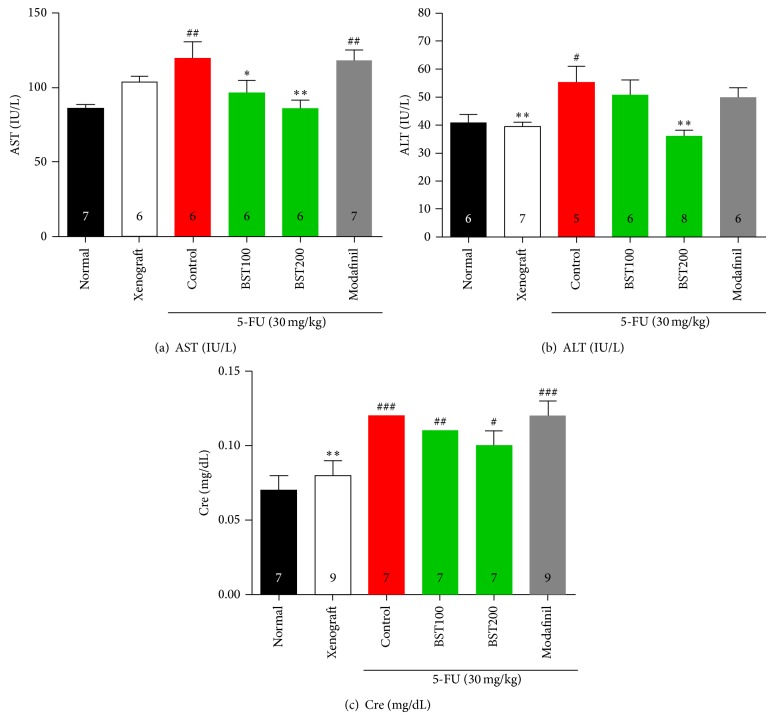
The effects of BST204 and modafinil with 5-FU treatment on AST (a), ALT (b), and Cre (c). Each value represents the mean ± S.E.M. ^#^ *P* < 0.05, ^##^ *P* < 0.01, and ^###^ *P* < 0.001 compared to the normal and ^*^ *P* < 0.05, ^**^ *P* < 0.01 compared to the control group. Naïve normal (normal); HT-29 cell inoculated + saline treated group (xenograft). Xenograft + 5-FU + vehicle treated group (control); xenograft + 5-FU + BST204-treated group (BST204). Xenograft + 5-FU + modafinil treated group (modafinil).

The levels of biochemical parameters were significantly different among the groups (Figures 5(a)–5(c)). The results indicated that serum levels of AST (Figure 5(a); *F*_5,32_ = 4.8, *P* < 0.01), ALT (Figure 5(b); *F*_5,32_ = 4.5, *P* < 0.01), and Cre (Figure 5(c); *F*_5,40_ = 5.4, *P* < 0.01) were significantly increased in the control group compared to the normal group. However, treatment with BST204 200 mg/kg markedly decreased levels of AST, ALT, and Cre compared to the control group.

**Figure 6:**
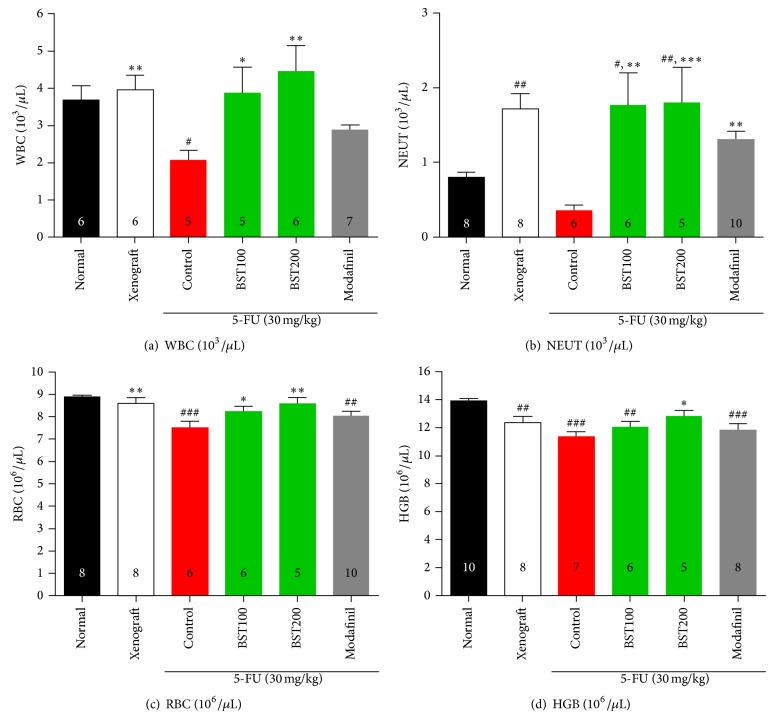
The effects of BST204 and modafinil with 5-FU treatment on WBC (a), NEUT (b), RBC (c), and Hgb (d). Each value represents the mean ± S.E.M. ^#^ *P* < 0.05, ^##^ *P* < 0.01, and ^###^ *P* < 0.001 compared to the normal and ^*^ *P* < 0.05, ^**^ *P* < 0.01, and ^***^ *P* < 0.001 compared to the control group. Naïve normal (normal); HT-29 cell inoculated + saline treated group (xenograft). Xenograft + 5-FU + vehicle treated group (control); xenograft + 5-FU + BST204-treated group (BST204). Xenograft + 5-FU + modafinil treated group (modafinil).

The levels of WBC (Figure 6(a); *F*_5,29_ = 3.3, *P* < 0.05), NEUT (Figure 6(b); *F*_5,37_ = 5.7, *P* < 0.01), RBC (Figure 6(c); *F*_5,39_ = 4.4, *P* < 0.01), and HGB (Figure 6(d); *F*_5,38_ = 6.7, *P* < 0.001) were significantly increased in the BST204 200 mg/kg treated group compared with control group. The levels of WBC were significantly reduced in 5-FU treated group (*P* < 0.05). However, in the BST treated groups the levels of WBC were dose-dependently increased (*P* < 0.05). The levels of RBC were significantly reduced in 5-FU treated group (*P* < 0.001). However, in the BST treated groups the levels of RBC were dose-dependently increased (*P* < 0.05). The levels of HGB were significantly reduced in 5-FU treated group (*P* < 0.001). However, the levels of HGB were increased in the BST high dose treated groups (*P* < 0.05).

### 3.4. The Tumor Volume

**Figure 7:**
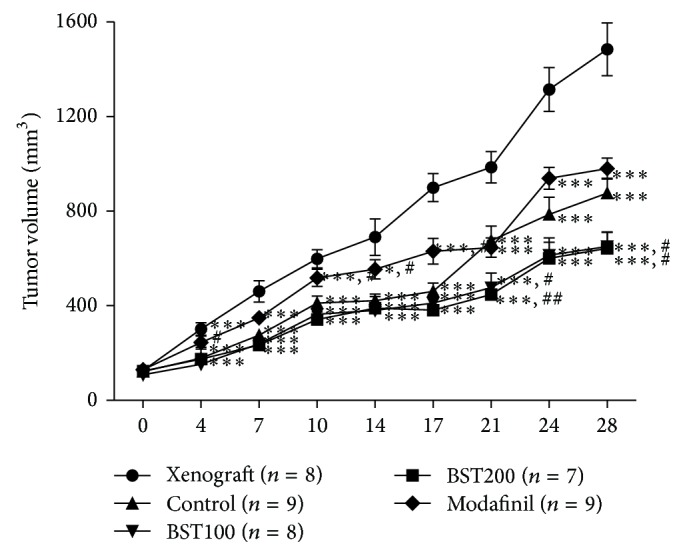
The effects of BST204 and modafinil with 5-FU treatment on tumor size. Each value represents the mean ± S.E.M. ^#^ *P* < 0.05, ^##^ *P* < 0.01, and ^###^ *P* < 0.001 compared to the normal and ^*^ *P* < 0.05, ^**^ *P* < 0.01, and ^***^ *P* < 0.001 compared to the control group. Naïve normal (normal); HT-29 cell inoculated + saline treated group (xenograft). Xenograft + 5-FU + vehicle treated group (control); xenograft + 5-FU + BST204-treated group (BST204). Xenograft + 5-FU + modafinil treated group (modafinil).

An ANOVA (5 × 9, tumor volume × time) performed on the tumor volume of the cancer revealed a significant group difference (*F*_5,32_ = 5.7, *P* < 0.01) on the effect of time (*F*_5,32_ = 5.7, *P* < 0.01) and a group × time interaction (*F*_5,32_ = 5.7, *P* < 0.01). Until the end of the experiment, the tumor volume in combined 5-FU and drug group was much smaller than that of xenograft group, as well as BST204 group and modafinil group (*P* < 0.05) (Figure 7). Tumor size is not significantly correlated with its cytokine, glycogen, and biochemical markers. 5-FU treatment reduced tumor size but its cytokine level was not affected.

## 4. Discussion

This study proved that the treatment with BST204 produced a significant decrease of physical fatigue in the running wheel activity and forced swimming test at the 27th day compared to that of the control group. Consistent with the behavioral data, BST204 markedly increased muscle glycogen activity and levels of WBC, NEUT, RBC, and HGB. Also it reduced 5-FU-induced TNF-*α*, IL-6, AST, ALT, and CRE levels, which are more effective than a positive control [47]. This result suggests that BST204 may improve chemotherapy-related fatigue and adverse toxic side effects.

5-Fluorouracil (5-FU) is a pyrimidine analog widely used as anticancer drug for different types of solid tumors [48–50]. Clinical use of 5-FU has been limited by its systemic toxicity [51–53], its tendency to induce drug resistance, and the difficulty of delivering and maintaining locally effective therapeutic concentrations [53–55]. Cancer related fatigue was induced by cancer itself or cancer chemotherapy.

Voluntary wheel running activity and swimming tests have been widely used as a measure of cancer related fatigue in animal models [8, 56–58]. 5-FU, a standard chemotherapy drug, is well known to reduce voluntary wheel running activity in mice. Decreases in voluntary activity and forced swimming test measured in the present study demonstrated that 5-FU chemotherapy produced fatigue in animal, consistent with a previous report [59] showing that the 30 mg/kg 5-FU significantly decreased such behaviors [56]. All animals were adapted to voluntary running wheel activity test for three days before drug treatment. We confirmed that wheel running activity measured during baseline among groups was not significantly different before the start of the experiment, as seen in Figure 2(a). These results suggest that the observed differences in VWRA among groups after drug may not influenced by group differences at the start of the experiment. In the current study, wheel running activity was assessed on 13th and 27th day of drug treatment for evaluation of fatigue, since previous and our pilot data demonstrated that tumor size after inoculation peaked on 4 weeks [60–62].

For assessing the VWRA, all mice were acclimated to the testing room for 5 minutes and examined running wheel activity for 10 minutes during the dark period. The pattern of wheel running activity among the experimental groups was very stable on the 13th and 27th day, respectively, as shown in Figure 2(b). Our results demonstrated that there were no significant differences and produced a very stable pattern among groups on days 13 and 27, suggesting that 5-FU can reduce voluntary wheel running activity in mice and this animal model is well established to evaluate the drug efficacy.

In our study, the running wheel activity and swimming time in the 5-FU treated control group were reduced compared to the normal group. However, treatment of BST204 significantly increased wheel activity and swimming time and glycogen synthesis of leg muscle similar to modafinil.

Chemotherapy drugs are toxins and may cause liver or kidney damage. Liver damage, also known as hepatotoxicity, causes the critical organ to underfunction. Also, high creatinine levels should be cautious when evaluating chemotherapy as a cancer treatment. It is reported that physiological changes of fatigue were closely related to increases in AST, ALT, and CRE levels, causing significant liver and kidney burden [63–65]. In the present study, AST, ALT, and CRE levels were measured in order to evaluate liver and kidney damage, respectively. 5-FU-induced levels of AST, ALT, and CRE were significantly reduced by treatment with BST204. Previous studies reported the preventive effect of ginsenoside Rg_3_ against hepatic and renal injury [66–71]. It has been shown that Rh_2_ has an antiapoptotic [72], anticancer effects [73] through inhibition of human hepatoma cell apoptosis [73]. Therefore, repeated administration of BST204, consisting of both components, Rg_3_ and Rh_2_, may have a synergistic effect for regulating cancer related fatigue.

In the cancer context, inflammation may be induced by common cancer treatments, including radiotherapy and chemotherapy. Proinflammatory cytokines (IL-1*β*, IL-6, IL-8, and TNF-*α*) are elevated in colorectal cancer (CRC), showing the presence of an active and permanent inflammatory state [74]. The rationale behind this includes the idea that cancer and chemotherapy can stimulate the release of peripheral proinflammatory cytokines. Cancer patients experience loss of appetite, pain, sleep disturbance, anorexia, and fatigue undergoing chemotherapy. The present study confirms that chemotherapy can increase levels of proinflammatory cytokines which can cause symptoms of fatigue, consistent with another animal study [75]. Similar to the current results, in animal models of sickness behavior, prolonged production of TNF-*α*, IL-6, and IL-1*β* can also lead to a variety of symptoms including fat and muscle wasting and behavioral changes similar to symptoms of depression that also occur in cancer patients [7–10]. The increase of proinflammatory cytokine coexists with psychological complaints, which can include depression and anxiety [76–79]. In the clinical studies, cancer patients and survivors will not be eligible for or interested in treatment with cytokine antagonists or other pharmacotherapies [80, 81].

Cancer related fatigue is a common problem undergoing treatment for cancer and may endure for months or years following completion of treatment in some patients. Fatigue has a negative impact on mood, social relationships, daily activities, and overall quality of life among both cancer patients and survivors. Despite its prevalence, the mechanisms underlying the onset and persistence of fatigue among cancer patients have not been determined. Although a variety of biological mechanisms have been proposed, the few studies to assess biological parameters (e.g., hematocrit, hemoglobin, albumin, and thyroid hormone) have typically not found a correlation with fatigue. Cancer related behavioral comorbidities such as fatigue, sleep disturbances, and depression have also been associated with inflammation, hypothalamic-pituitary-adrenal (HPA) axis dysregulation, and other neuroendocrine changes [82]. In particular, proinflammatory cytokines, IL-1*β*, IL-6, and TNF-*α* may be released as part of the host response to the tumor or in response to tissue damage or depletion of immune cell subsets associated with cancer treatment [83, 84]. Some investigators report elevated plasma levels of certain proinflammatory cytokines [85–87], for example, TNF-*α* [88, 89], supporting a role for cytokine driven inflammation. Recently, many studies reported the remedy effect of herbal medicine.* P. ginseng* is a popular herbal remedy that has been used in eastern Asian countries for treatment of various health-related complaints [90–93]. Also, ginsenosides have been reported to exhibit various biological activities, including antiallergic [94, 95], anti-inflammatory action [96], and antitumor effect [97–101]. A clinical study also proved antifatigue effects of panax ginseng Meyer [20]. Previous studies reported that Rg_3_ has the antioxidant effect and promotes the immune response in the mice [102–104]. Rh_2_ can inhibit inflammatory cytokines in the LPS-induced raw 264.7 macrophage cell line and microglia [105–108]. Consistent with previous studies, we found that the administration of BST204 inhibited secretion of proinflammatory cytokines, TNF-*α* and IL-6. These results support the idea that the inhibitory effect of BST204 against CRF is mediated through inhibition of inflammatory systems.

Many of cancer patients experience anemia, which may contribute to CRF. Also, a study of patients diagnosed with colorectal cancer, lung cancer, or ovarian demonstrated a correlation between increased symptoms of fatigue and abnormally low levels of hemoglobin, especially during chemotherapy. The present study examined this possibility and demonstrated that treatment of BST204 recovered 5-FU-induced hematosuppression, evidenced by increasing hematologic parameters, WBC, NEUT, RBC, and HGB. The results of the present study indicate that anemia may develop in response to 5-FU and may contribute CRF in the present mice model. The biological factors include lower levels of red [109] and white blood cells, cytokine dysregulation, lower hemoglobin levels, cancer-related treatments [110], and disease status [111]. However, the present study demonstrated that BST204 significantly prevented 5-FU-induced anemia, suggesting that the inhibitory effects of BST204 on CRF can be explained by its promotion of hematopoiesis. However, further studies are needed to examine more precise mechanisms underlying enhancing effects of BST204 on hematopoiesis.

One of the most prescribed drugs for management of CRF is modafinil. Modafinil is a stimulant with a selective site of action in the brain that is better tolerated than other drugs [1]. However, adverse effects of modafinil treatment were reported such as headache, infection, nausea, nervousness, anxiety, and insomnia, all of which were generally mild [2]. In the present study, modafinil was used as a positive control in order to compare its efficacy with BST204. It has been shown that BST204 was more effective in reducing CRF responses than modafinil treatment.

These findings suggest that BST204 may improve cancer related fatigue via regulation of inflammatory responses and hematopoiesis. Further research is needed to develop a better understanding of pathology of CRF and to discover effective interventions to preserve muscle mass and reduce fatigue related responses in mice.
